# Compromised Muscle Properties in a Severe Hypophosphatasia Murine Model

**DOI:** 10.3390/ijms242115905

**Published:** 2023-11-02

**Authors:** Emily G. Pendleton, Anna S. Nichenko, Jennifer Mcfaline-Figueroa, Christiana J. Raymond-Pope, Albino G. Schifino, Taylor M. Pigg, Ruth P. Barrow, Sarah M. Greising, Jarrod A. Call, Luke J. Mortensen

**Affiliations:** 1 Regenerative Bioscience Center, Rhodes Center for ADS, University of Georgia, Athens, GA 30602, USA; 2 Department of Physiology & Pharmacology, University of Georgia, Athens, GA 30602, USA; 3 School of Kinesiology, University of Minnesota, Minneapolis, MN 55455, USA; 4 School of Chemical, Materials and Biomedical Engineering, University of Georgia, Athens, GA 30602, USA

**Keywords:** hypophosphatasia, mitochondria, muscle, bone, musculoskeletal disease, metabolic bone disorders

## Abstract

Hypophosphatasia (HPP) is a rare metabolic bone disorder characterized by low levels of tissue non-specific alkaline phosphatase (TNAP) that causes under-mineralization of the bone, leading to bone deformity and fractures. In addition, patients often present with chronic muscle pain, reduced muscle strength, and an altered gait. In this work, we explored dynamic muscle function in a homozygous TNAP knockout mouse model of severe juvenile onset HPP. We found a reduction in skeletal muscle size and impairment in a range of isolated muscle contractile properties. Using histological methods, we found that the structure of HPP muscles was similar to healthy muscles in fiber size, actin and myosin structures, as well as the α-tubulin and mitochondria networks. However, HPP mice had significantly fewer embryonic and type I fibers than wild type mice, and fewer metabolically active NADH+ muscle fibers. We then used oxygen respirometry to evaluate mitochondrial function and found that complex I and complex II leak respiration were reduced in HPP mice, but that there was no disruption in efficiency of electron transport in complex I or complex II. In summary, the severe HPP mouse model recapitulates the muscle strength impairment phenotypes observed in human patients. Further exploration of the role of alkaline phosphatase in skeletal muscle could provide insight into mechanisms of muscle weakness in HPP.

## 1. Introduction

Hypophosphatasia (HPP) is a heritable metabolic disorder characterized by mutations in the *ALPL* gene that encodes tissue non-specific alkaline phosphatase (TNAP), an enzyme found in a variety of tissues including kidneys, liver, brain, and bone [1]. There are more than 300 known mutations in the *ALPL* gene, and HPP has a range of symptoms and pathologies, with more severe forms of the disease presenting earlier in life [2]. HPP is a rare disease, with an estimated prevalence of severe cases ranging from 1/100,000 [3] to 1/300,000 [4], moderate cases at 1/6370 [4] in Europe, and recent estimates of mild case prevalence ranging from 1/3100 [5] throughout Europe to 1/1692 in Spain [6]. A mutated *ALPL* gene leads to the accumulation of pyridoxal-5′-phosphate and inorganic pyrophosphate, both of which are natural substrates for TNAP. The buildup of inorganic pyrophosphate in particular inhibits the mineralization process; thus, HPP tends to have the primary effect of rickets in children and osteomalacia in adults [7]. A majority of adult patients with HPP report a need for assistive mobility devices due to an unusual gait; and 62% self-report muscle weakness [8].

The presentation of muscle weakness is poorly understood across all forms of HPP—in utero, perinatal, infantile, childhood, and adult [9]. In the infantile and childhood form, muscle weakness can cause developmental delays in walking or other gross motor milestones and contributes to a characteristic waddling gait [10]. Adult onset HPP patients can exhibit debilitating muscle weakness [11] and muscle fatigue [9]. Muscle biopsy specimens during early childhood development of HPP suggest isolated muscle fiber structural abnormalities and small muscle fiber sizes; however, this interpretation is limited by the nature of the work (i.e., non-experimental case studies) [10].

Over the past few years, a human recombinant TNAP enzyme replacement with a bone-targeting peptide sequence marketed as Asfotase Alfa has begun to rescue patients with severe HPP and improve the quality of life for those with more mild symptoms. In addition to its direct bone effects, clinical studies have suggested that Asfotase Alfa improves motor function in children, adolescents, and adults who suffered from infantile or childhood HPP [12,13,14,15]. Muscle and bone strength are biomechanically linked, and so it is possible that muscle weakness in HPP is caused by weakness in associated bones or decreased loading from the sedentary lifestyle associated with HPP. Some provocative recent case studies have found substantial improvements in muscle-related measures like grip strength or walking strength within a few months of treatment, even with small or undetectable alterations in bone quality measures [16,17,18]. Therefore, contributions to HPP muscle weakness that can be reversed quickly, like diminished ALP activity in the muscle or associated biochemical signaling, likely play a larger role than biomechanics or underlying muscle developmental issues. A genetically engineered sheep model of HPP was created to better elucidate the mechanisms of HPP pathophysiology, and the model accurately replicated low levels of serum ALPL, low bone mineral density, and impaired gait [19]. Qualitative assessment of muscle biopsy specimens from the sheep model also suggested smaller muscle fiber sizes and abnormal mitochondrial structures, which has been supported by grip strength and balance testing in the homozygous ALP knockout HPP mouse model and abnormal in vitro mouse progenitor cell mitochondrial function [20]. However, detailed functional measurements of muscle contractile properties and muscle mitochondrial respiration remain unreported in the more widely used mouse model of HPP.

The primary objective of this study was to quantitatively assess skeletal muscle function and structure in a murine model of severe HPP. Determining the functional properties of skeletal muscle from HPP could advance the use of mouse models to better understand the human muscle weakness pathology and broaden our knowledge of muscle–bone relationships.

## 2. Results

### 2.1. Quantitative Assessment of Overall Phenotype in HPP Mice

The congenital HPP mouse model we used in this work is a homozygous knockout of tissue non-specific alkaline phosphatase (*Alp*^−/−^*)*. In line with numerous prior studies for the *Alp*^−/−^ HPP model [21,22], we found impairment in bone mineralization at days 10–14. For this muscle-focused study, we monitored disease progression in *n* = 31 mice using weight, body condition score [23], and behavioral metrics (e.g., hunched posture, irritability, isolation, abnormal movement, and ruffled fur) longitudinally on days P10, P12, and P14 (Table 1). On average, HPP mice gained weight during this evaluation period, with a trend towards a smaller size than their healthy WT littermates. The HPP mouse body condition was within a healthy range (Table 1). Observations of behavior were recorded on these days, and since only abnormal movement was observed, it is included in Table 1. At P10, 9.7% of the HPP population had abnormal movements, which increased to 19.3% by P12, and 35.5% by P14. Overall, the HPP mice remained adequately healthy at P14 for experimental use.

### 2.2. Muscle Size and Contractile Function

**Figure 1:**
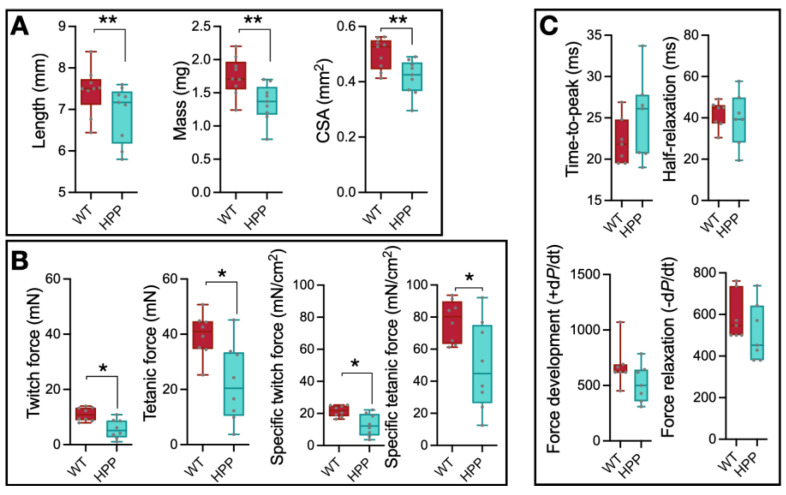
Muscle contraction analysis in hypophosphatasia (HPP) and wildtype (WT) muscles. (**A**) EDL muscle length, mass, and physiological cross-sectional area (CSA) were greater in WT compared to HPP mice (**, *p* ≤ 0.01; *n* = 9 mice/group). (**B**) EDL muscle contractile twitch and peak isometric forces were lower in hypophosphatasia (HPP) mice compared to WT even when accounting for CSA (i.e., specific twitch and specific tetanic force) (*, *p* ≤ 0.05; *n* = 8 mice/group). (**C**) There were no statistically significant differences in twitch contractile properties (time-to-peak and half-relaxation time) nor tetanic contractile properties (force development rate and force relaxation rate) between WT and HPP mice (*p* ≥ 0.131; *n* = 7 mice/group). Independent *t*-tests assessed mean differences between WT and HPP genotypes.

The EDL muscles from WT and HPP mice were excised immediately after euthanasia and assessed for size and in vitro contractility. Physiological cross-sectional area was calculated for each muscle based on length, mass, and pennation angle [24,25]. EDL muscle length, mass, and physiological cross-sectional area were significantly less in HPP compared to WT mice (Figure 1A, *p* ≤ 0.01). Muscle twitch (the force generated from a muscle contraction in response to a single action potential) and peak isometric tetanic (the maximum force generated from a sustained muscle contraction) forces were significantly less in HPP muscle compared to WT (Figure 1B, *p* ≤ 0.05), as well as the specific twitch and tetanic contractile force (i.e., normalized to physiological cross-sectional area) (Figure 1B, *p* ≤ 0.05), suggesting an underlying contractile issue related to excitation–contraction coupling and/or cross-bridge formation. Twitch contractile properties (time-to-peak and half-relaxation time) and tetanic contractile properties (force development rate and force relaxation rate) were assessed from force–time tracings, and there were no significant differences between groups (Figure 1C, *p* ≥ 0.131).

### 2.3. Mitochondrial Function

**Figure 2:**
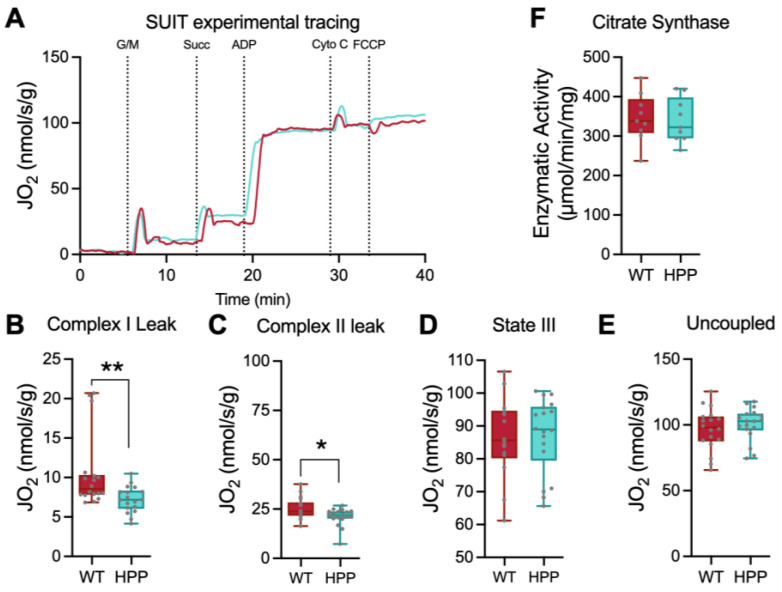
Respiration of permeabilized fiber bundles in hypophosphatasia (HPP) compared to wild type (WT) mice. (**A**) Representative permeabilized fiber bundle mitochondrial respiration from WT (red line) and HPP (cyan line) mice. (**B**) Complex I leak respiration, obtained by the addition of glutamate/malate in the absence of ADP, was lower in HPP compared to WT (**, *p* ≤ 0.01; *n* = 18 samples/group). (**C**) Complex I/II leak respiration, obtained by the addition of glutamate/malate/succinate in the absence of ADP, was lower in HPP compared to WT (*, *p* ≤ 0.05; *n* = 18 samples/group). (**D**) State III respiration obtained in the presence of glutamate/malate/succinate and ADP was not different between WT and HPP (*p* = 0.982; *n* = 18 samples/group). (**E**) Uncoupled respiration initiated by the addition of FCCP was not statistically different between HPP and WT (*p* = 0.425). (**F**) Citrate synthase activity, a marker of skeletal muscle mitochondrial content, was not statistically different between HPP and WT (*p* = 0.819; *n* = 9 mice/group). Independent *t*-tests assessed mean differences between WT and HPP genotypes.

The TA muscles from WT and HPP mice were isolated immediately after euthanasia and prepared for mitochondrial respiration assessment (Figure 2A). Complex I-supported leak respiration (malate, glutamate, no ADP) (Figure 2B, *p* ≤ 0.01) and complex II-supported leak respiration (malate, glutamate, succinate, no ADP) (Figure 2C, *p* ≤ 0.05) were significantly lower in muscle from HPP mice compared to WT; however, after the addition of supra-physiological levels of ADP to induce state III respiration, these differences disappeared (Figure 2D, *p* = 0.982). Furthermore, uncoupled respiration rates obtained by titration of FCCP were not significantly different between the groups (Figure 2E, *p* = 0.425). The enzyme kinetics for citrate synthase were assessed as an indicator of skeletal muscle mitochondrial content, and there were no significant differences in activity between HPP and WT (Figure 2F, *p* = 0.819). After normalizing by citrate synthase activity, the same trends in mitochondrial activity were maintained, with significant differences between WT and HPP basal activity rate (*p* = 0.003) and complex I leak respiration (*p* = 0.005). We found no difference in citrate synthase-normalized complex I-and II-derived leak respiration (*p* = 0.080), normal state III respiration (*p* = 0.752), and normal uncoupled respiration (*p* = 0.523).

**Figure 3:**
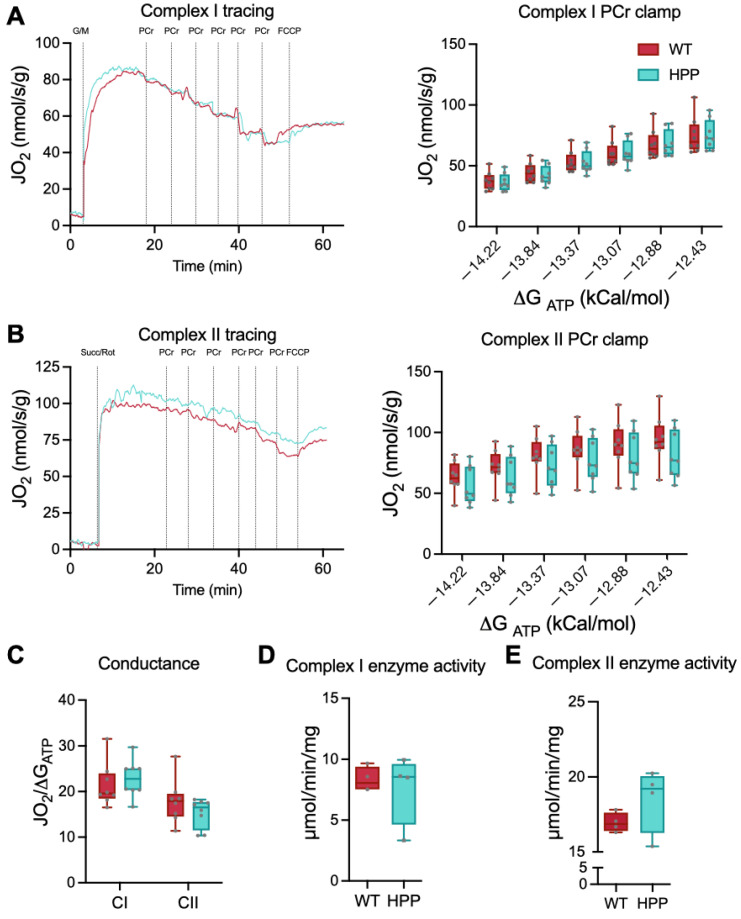
Interrogation of complex I- and complex II-specific permeabilized fiber bundle respiration, electron conductance, and enzyme activities. (**A**) Complex I-supported (malate/glutamate) creatine kinase (CK) clamped respiration was not different between hypophosphatasia (HPP, cyan line) and wildtype (WT, red line) muscle at any clamped ATP re-synthesis demand state (*p* = 0.753; *n* = 8 samples/group). (**B**) Complex II-supported (rotenone/succinate) CK clamped respiration was not different between HPP and WT muscle at any clamped ATP re-synthesis demand states (*p* = 0.183; *n* = 8 samples/group). (**C**) Electron conductance for complex I- and complex II-supported respiration was calculated from the slopes of the respiration (*J*O_2_) and clamped energy state (ΔGATP) relationships and were not different between WT and HPP (*p* ≥ 0.229; *n* = 8 samples/group). (**D**,**E**) Complex I and complex II enzyme activities were not statistically different between WT and HPP muscle (*p* ≥ 0.254; *n* = 4 samples/group). Independent *t*-tests assessed mean differences between WT and HPP genotypes.

Based on the initial findings of differences in complex I and II leak respiration rates, we conducted a more sensitive respiration test to detect electron conductance through the electron transport chain with complex I or II substrates. The test described by Fisher-Wellman et al. [26,27] and employed by our group previously [28] uses the enzymatic reaction of creatine kinase (CK) and phosphocreatine (PCr) to stepwise “clamp” ATP/ADP ratios at different ATP free energies such that a high demand for ATP re-synthesis is −12.8 ∆G_ATP_ and a low demand for ATP re-synthesis is −14.8 ∆G_ATP_. As described by Fisher-Wellman et al. [26], when ADP phosphorylation (ATP re-synthesis) is strongly coupled to respiration (i.e., oxygen consumption), then *J*O_2_ is expected to be titrated down proportionately to a rise in ∆G_ATP_. Using this PCr CK-clamp technique, we found that respiratory flux (*J*O_2_) diminished with greater ATP free energies in both HPP and WT, i.e., more negative ∆G_ATP_, greater ATP/ADP ratio (Figure 3A,B). These data agree with the theoretical and experimental relationship reported between ATP free energy and *J*O_2_ in other tissues and establishes the CK clamp technique as a valid tool for the assessment of mitochondrial bioenergetics in muscle from HPP model mice. In agreement with the state III and uncoupled respiration results above, there were no significant differences in maximal respiration rates (−12.8 ∆G_ATP_) between groups (*p* ≥ 0.183).

The linear phase of the CK clamp relationship between *J*O_2_ and ∆G_ATP_ had been used previously to define electron conductance (i.e., ease of flow) through the electron transport chain and reflects mitochondria sensitivity to changing energetic demands. There were no significant differences between HPP and WT muscles in either complex I or complex II electron conductance (Figure 3C, *p* ≥ 0.229).

Finally, the enzymatic activities of complex I and complex II were individually assessed to determine if impaired enzyme function contributed to the leak respiration differences between HPP and WT muscles. There were no significant differences between HPP and WT muscles for either complex I or complex II activities (Figure 3D,E, *p* ≥ 0.254).

### 2.4. Muscle Histological Characteristics

**Figure 4:**
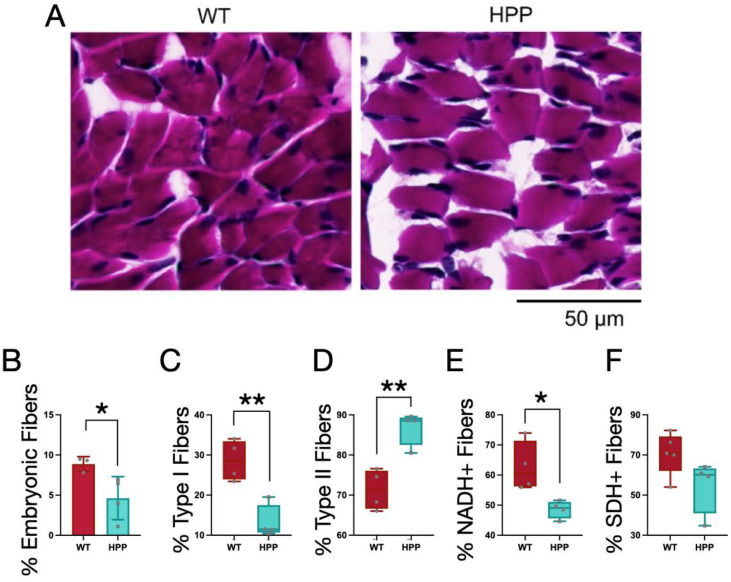
Effect of hypophosphatasia (HPP) on muscle fiber morphology and type. (**A**) Hematoxylin and eosin staining of gastrocnemius muscles from wildtype (WT) (*n* = 5) and HPP (*n* = 4) mice reveal normal muscle fiber structure. (**B**) HPP mice (cyan) had a significantly decreased proportion of embryonic MyHC-expressing muscle fibers compared to WT (red) (*, *p* ≤ 0.05; *n* = 3–4/group), with both (**C**) significantly fewer type I (or slow) muscle fibers (**, *p* ≤ 0.01; *n* = 4/group) and (**D**) significantly more type II (or fast) muscle fibers (**, *p* ≤ 0.01; *n* = 4/group). (**E**) As a percentage of the total muscle fibers, there were significantly fewer NADH-positive fibers in the HPP mice (*, *p* ≤ 0.05; *n* = 4/group), (**F**) but there was no significant difference in the percent of SDH-positive fibers between the groups (*p* = 0.087, *n* = 4/group). Independent *t*-tests assessed mean differences between WT and HPP genotypes.

To further investigate the murine HPP model muscle pathology and explore the mechanisms for the contractile and mitochondrial functions described above, several histological and multi-photon imaging tests were conducted post-mortem. Using histological sections, muscle fiber types within the gastrocnemius (GA) muscle were evaluated. Fibers from both the WT and HPP mice had peripheral nuclei, i.e., no evidence of centralized nuclei (often indicative of muscle fiber damage; Figure 4A). At P14, murine skeletal muscles are still developing, so the expression of embryonic (developmental) MyHC was evaluated. HPP mice had a lower percentage of total fibers staining positive for embryonic MyHC compared to WT (Figure 4B, *p* ≤ 0.05). In terms of muscle fiber type distributions, HPP mice had a greater proportion of fast (MyHC_fast_) and less slow (MyHC_slow_) fibers compared to WT (Figure 4C,D, *p* ≤ 0.01).

Histological sections of the GA muscles from HPP and WT mice were also stained for NADH and SDH as markers of muscle fiber oxidative capacity. The fibers from HPP mice had a lower amount of fibers positive for NADH (Figure 4E, *p* ≤ 0.05) and a trend towards a lower amount of fibers positive for SDH (Figure 4F, *p* = 0.087). NADH and SDH stains complement complex I and complex II activity readouts, respectively, and these findings warrant further development of a model of metabolic function and content in the context of HPP disease etiology.

### 2.5. Muscle Morphological Characteristics

**Figure 5:**
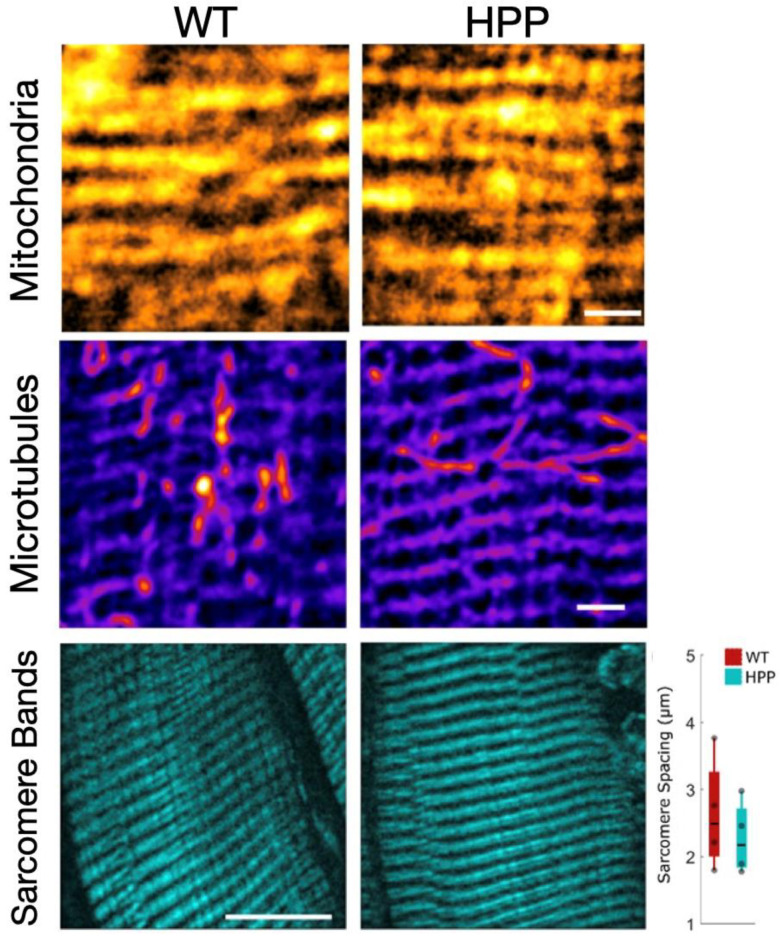
Sarcomere α-tubulin networks are similar in wildtype (WT) and hypophosphatasia (HPP) muscles. (**Top**) TA muscles were treated with Mitospy Orange to visualize the mitochondrial network organization. In both WT and HPP muscles, the mitochondrial network took on similar linear organization. Scale bar: 2 µm (**Center**) The TA muscle α-tubulin networks were visualized by antibody staining. The microtubules of the WT and HPP muscles appear similar to one another, with both displaying rectilinear organization. (**Bottom**) The basic contractile subunit of muscles is the sarcomere, consisting of actin and myosin filaments between the z-line. Using SHG imaging, the sarcomere structures appeared to be similar between the WT and HPP groups. To determine if there were more sarcomeres in the WT than the HPP muscle, we determined the distance between the myosin bands and found no significant difference in the myosin band spacing between the groups (*p* = 0.105, *n* = 4/group). Independent *t*-tests assessed mean differences between WT and HPP genotypes. Scale bar: 10 µm.

Multi-photon microscopy was used to evaluate the morphological attributes of P14 TA fibers from HPP and WT mice. The mitochondrial network was evaluated by fixing the fibers and staining with MitoSpy Orange (Sony Biotechnology Inc., San Jose, CA, USA). The mitochondria in the WT and HPP muscle had qualitatively similar network structures (*n* = 6 mice/group; Figure 5(top)). Both genotypes had conserved regular arrangements of mitochondrial networks and it does not appear that HPP disrupts the spatial relationship of mitochondria.

Finally, fibers are able to maintain their shape and function using actin filaments and microtubules [29]. Alterations to the microtubule cytoskeleton have been proven to contribute to the muscle defects in diseases such as Duchenne Muscular Dystrophy [30]. Therefore, we used α-tubulin staining to determine if the microtubule network of the HPP skeletal muscle was disrupted (Figure 5(center)). The microtubule grid was conserved in HPP mice, with regularly spaced longitudinal and lateral microtubules (*n* = 5 mice/group), and a similar density of the subsarcolemma. Therefore, we concluded that microtubule structures in the skeletal muscles of the HPP mice are not impaired by disease.

Next, the sarcomere structure and spacing was evaluated. The most widely accepted model of muscle contraction involves actin filaments sliding past the myosin filaments to contract the sarcomere between the lateral boundaries of the sarcomere, the Z-line. These contractile myofilaments have a regular linear structure that provides intrinsic contrast with second harmonic generation (SHG). To determine if the difference in force generated between HPP and WT muscles could be related to a difference in sarcomere spacing, we examined the actin and myosin structures of the sarcomeres within the TA muscle using SHG imaging. The sarcomere structure and the distance between the sarcomere groups was not found to be different between WT and HPP (Figure 5(bottom), *p* = 0.105), and so we expect a similar number of sarcomeres within any given area.

## 3. Discussion

This study comprehensively evaluated skeletal muscle function in a murine model of HPP at postnatal day 14. Metabolic bone disorders often have wide-reaching effects throughout the musculoskeletal system [31], many of which may directly or indirectly impact skeletal muscle. Therefore, although skeletal muscle weakness is a frequent co-morbidity with metabolic bone disorders [32,33,34], it can be challenging to parse out the relative contributions to muscle weakness that arise from a host of factors such as direct genetic defects [35], hormone and ion imbalances [36], biomechanical effects [37], neurological issues [38], or associated sedentary lifestyles [33,39]. In contrast to the prior consensus [19,22], we found that muscles in a pre-morbid developmental HPP mouse model were small and disproportionately weak (i.e., low specific force), with mild mitochondrial dysfunction, and histological features suggesting altered or accelerated muscle development.

HPP patients typically have a smaller bone mass and muscle mass than healthy counterparts, and experience waddling gaits, difficulty moving off of the floor, muscle stiffness and pain [11,40]. The cause of this muscular weakness is currently poorly understood, and is not well correlated with skeletal disease severity, as it is present across the spectrum of human HPP disease. Therefore, animal models of the HPP disease can offer us a chance to better elucidate muscle pathophysiology. Murine models are effective for studying the craniofacial abnormalities and abnormal bone formation associated with HPP at various levels of disease severity [22,41,42]. Moving forward, the *Alpl*^−/−^ severe HPP murine model used in this work that is characterized by early muscle weakness is a good candidate to better understand muscular consequences of HPP in humans and muscle response to asfotase alpha therapy.

Mammalian skeletal muscle is a heterogenous composition of slow- and fast-twitch motor units, and thus muscle fibers are commonly characterized by contractile, histological, and/or biochemical properties. Histological fiber types are often delineated by MyHC protein expression [43], as was used herein, but can also be defined by oxidative capacity, e.g., staining for SDH [44]. At P14, gastrocnemius (GA) muscle fibers of WT mice were ~71% type II (fast) and 29% type I (slow). These values are consistent with the MyHC developmental literature of mice; specifically, GA muscle, or hindlimb muscles in general, are reported to have between 30 and 40% fast fibers between P7–P10, and this increases to a 90% fast fiber type composition by P20 [45,46]. While the fiber type distribution in muscle from HPP mice at P14 also fell within this range (i.e., 89% fast, 11% slow), the percentages were significantly different from WT, and potentially closer to the predominant fast phenotype observed at P20 in the literature for genetically WT mice. This could indicate an accelerated development process in the skeletal muscle of HPP mice; however, the nuanced process of mammalian skeletal muscle fiber type development is influenced by myoblast fusion, innervation, and hormonal regulation (reviewed in [47]).

During embryonic stages of mouse development, primary myotubes are observed first and express embryonic, neonatal, and a form of slow MyHC. However, second generation myotubes, formed later in utero, will eventually become the permanent adult fibers and primarily express embryonic, neonatal, and fast MyHC (reviewed in [48]). During postnatal development, these fiber types will be influenced by nerve innervation and recruitment patterns such that highly recruited fibers will take on predominately slow-twitch attributes and a MyHC composition. The literature indicates that embryonic MyHC goes from ~16–20% expression between P7–P10 to 0% expression by P20 as it is replaced by mature MyHC isoforms [45,46]. Both the WT and HPP data from this study are in line with this developmental trend, with statistically less expression of embryonic MyHC at P14 in HPP compared to WT muscles. This agrees with the higher expression of fast MyHC in HPP mice; however, the cause of this potential accelerated developmental time course is unclear. The finding of accelerated aging in HPP muscles is consistent with an increased aging phenotype found in the long bones and skeletal stem cells of a mild HPP mouse model [49].

Considering the greater expression of fast MyHC, several contractile and morphological properties of HPP muscle are intriguing. The absolute force was 43% less in HPP muscles and the contractile properties of muscle force development and relaxation were not different from WT. Predominately fast muscle fibers are characterized by high force contractions and high rates of force production. The in vitro contractile approach utilized here by-passes motor unit recruitment and the neuromuscular junction [50], so central and peripheral nerve activation patterns that may be important in clinical disease phenotype are not responsible for the contractile weakness observed in our work. Furthermore, while HPP muscles were smaller by 23% on average, the absolute contractile forces were impaired even after compensating for size differences. When there are discrepancies in specific force, this can often indicate a physiological issue with force-generating or force-transmitting structures of the muscle fiber. Our analysis of the sarcomere and cytoskeletal protein structure, though not comprehensive, did not strongly indicate abnormalities in the structure of force-generating (i.e., sarcomere) and/or force-transmitting (i.e., cytoskeletal proteins) networks. This could implicate either excitation–contraction coupling and/or cross-bridge formation as potential areas to further investigate for the mechanism of contractile weakness in HPP.

Upon early indication of complex I and complex II leak state deficits in HPP mice, we conducted a comprehensive evaluation of both complex I and II electron conductance and enzyme kinetics and detected no significant differences compared to the WT littermates. Furthermore, the mitochondrial network organization did not appear to be abnormal using multi-photon microscopy. This does not necessarily contradict the work by Williams et al. in which electron microscopy of muscle biopsy specimens of a HPP sheep model revealed abnormal structures in the mitochondrial reticulum [19]. First, although the authors noted muscle weakness observationally, they identified abnormalities in the mitochondria ultrastructure by electron microscopy and did not explore quantitative mitochondrial or functional properties of the muscle that were the focus of our work. Indeed, our analysis of mitochondrial structure may not be able to resolve the differences in reticular structure found by EM. However, reticular misfolding would be expected to impact mitochondrial function, which was not a strong observation in this work. Since our specific study design focused on HPP early in the animal lifespan, it is possible that in non-lethal HPP, mitochondrial structural alterations could be caused by disease pathology (e.g., consistent exposure to an imbalanced mitochondrial state as observed in other work by Zhang et al. [20]) and a more severe mitochondrial pathology may have emerged with time. One potential mechanism is supported by the function of tissue non-specific alkaline phosphatase (TNAP) as a nucleotidase that hydrolyzes ATP, ADP, and AMP [51]. Reduced activity of TNAP causes a buildup of extracellular and intracellular ATP [20], which has been found to be associated with accelerated aging and senescence [49], and more broadly with systemic inflammation [52]. It is possible that in our study, isolating mitochondria from the diseased environment to test their oxygen consumption ameliorates the energetic imbalances (e.g., excess ATP) that occur in vivo and would, over time, lead to the permanent mitochondrial damage observed in the sheep model. Therefore, it is premature to conclusively rule out a metabolic deficit contributing to the muscle pathology observed in HPP.

In conclusion, the HPP mouse model used herein demonstrated muscle weakness similar to that reported in patients with HPP, and both the severe and mild HPP mouse models should be further evaluated to elucidate the role of *Alpl* and its protein product TNAP on muscle fiber development. Furthermore, greater resolution of the disease pathology by investigating earlier and later development ages around postnatal day 14, and in less severe adult models of HPP, will likely help further define contractile and metabolic pathologies that exist with this disease. While the muscle pathology of HPP is a secondary concern clinically, considering the important role of muscles providing the primary bending movements on bones, it remains critical to better understand the contractile weakness in HPP and the extent to which the muscle can respond appropriately to rehabilitation. These future endeavors hold promise for improving the quality of life for patients with HPP.

## 4. Materials and Methods

### 4.1. Animals

This study used *Alp*^−/−^ mice, a commercially available mouse line with homozygous knockout of tissue non-specific alkaline phosphatase gene (Jackson Labs, Bar Harbor, ME, USA) [22]. The mouse line has negligible levels of tissue non-specific alkaline phosphatase activity in the body, which causes postnatal deficiencies in mineralization throughout the skeleton [21,22]. The *Alp*^−/−^ mouse skeletal phenotype mimics infantile HPP, including elevated levels of inorganic pyrophosphate together with the bone mineralization impairment. The mice develop epileptic seizures and apnea [42], which can be delayed by pyridoxine supplementation using modified laboratory rodent diet 5001 with 325 ppm pyridoxine (TestDiet, Richmond, IN, USA) to extend their lifespan to 18–22 days [53]. Knockout *Alpl*^−/−^ mice were identified by PCR at birth (Day 0) in accordance with a protocol developed by the Jackson Laboratory (Bar Harbor, ME, USA). Knockout *Alpl*^−/−^ mice are referenced moving forward as HPP mice and their wildtype littermates as WT mice.

### 4.2. Study Design

All experimental procedures were conducted on HPP and WT mice at P14 (postnatal day 14). The P14 timepoint was based on our previous work with this mouse model. In our hands, the *Alpl*^−/−^ mice start to succumb to complications of HPP by P18 with all HPP mice deceased by P22 [22,53]. One or two days prior to death, the mice begin to experience a decline in body mass. We therefore decided to use P14 to examine skeletal muscle pathology prior to severe pathological onset and contra-indicating factors such as loss of body mass.

To rapidly assess the health of the HPP mice, we monitored the body condition, weight, and behavior of HPP mice on days P10, P12, and P14. Body condition ranged on a scale from 1–5, with 1 representing an emaciated mouse and a score of 5 indicating an obese mouse [23]. The well-conditioned body score of 3 is used to describe a healthy mouse. Mice were euthanized and omitted from this experiment if their body condition was 2 or less. The weights of HPP mice were recorded along with the weights of their WT littermates. Since litter size can impact the weight of littermates, we compared HPP mice weights to the average weights of the healthy littermates. The behavior of the HPP mice was evaluated at each timepoint as well. Binary observations of hunched posture, irritability, isolation, abnormal movement, or ruffled fur were recorded. If a total of 3 or more abnormal behaviors were noted, mice were euthanized and excluded from the study. The WT littermates of the HPP mice included here had normal body conditions, age-appropriate weights, and no abnormal behaviors. All work was performed after approval by the University of Georgia Institutional Animal Care and Use Committee.

At P14, extensor digitorum longus (EDL) muscles from HPP and WT mice were excised and tested for muscle contractile function in vitro. The in vitro approach was used because preliminary testing of an in vivo approach proved too difficult due to the small size of the animals. The EDL muscle was selected because both distal and proximal tendons were accessible and it was slightly larger than the soleus muscle (an alternative muscle often used for in vitro contractility). The tibialis anterior (TA) muscle was used for mitochondrial respirometry experiments because it was large enough to isolate several fiber bundles for biological replicates, as opposed to the EDL that was sometimes less than 1 mg in size. The TA muscle was also used to assess sarcomere spacing, mitochondrial network, and cytoskeletal protein organization using two-photon microscopy. Finally, the gastrocnemius (GA) muscle was isolated and saved for histological assessment of fiber type. The GA, like the TA and EDL muscles, is a lower limb skeletal muscle highly utilized during ambulation.

### 4.3. Experimental Methodology

#### 4.3.1. Muscle Isolation and Preparation for Mitochondrial Experiments

Immediately following sacrifice, the TA muscles from HPP and WT animals were dissected on a chilled aluminum block in 4 °C Buffer X (7.23 mM K_2_EGTA, 2.77 mM Ca K_2_EGTA, 20 mM imidazole, 20 mM taurine, 5.7 mM ATP, 14.3 mM PCr, 6.56 mM MgCl_2_-6H_2_O, 50 mM k-MES). Muscles were carefully dissected into bundles and permeabilized with incubation on a rocker in Buffer X and saponin (50 μg/mL) at 4 °C for 30 min. Following permeabilization, muscle fiber bundles were rinsed for 15 min in Buffer Z (105 mM k-MES, 30 mM KCl, 10 mM KH_2_PO_4_, 5 mM MgCl_2_, 0.5 mg/mL BSA, 1 mM EGTA) at 4 °C. All respiration experiments were carried out using a Clarke-type electrode on an Oroboros O2K-Oxygraph (Oroboros, Innsbruck, Austria).

#### 4.3.2. In Vitro Assessment of Muscle Function

Live EDL muscles were excised, measured for weight and length, and used to assess contractile properties as previously described [54]. Muscle weight and length data, using a fiber length-to-muscle length ratio of 0.44 was used to calculate physiological cross-sectional area for the determination of specific P_o_. Briefly, EDL muscles were secured onto a dual-mode muscle lever system (300B-LR; Aurora Scientific Inc., Aurora, ON, Canada) inside a glass chamber filled with 0.38 mL of Krebs Ringer bicarbonate buffer. The buffer was maintained at 25 °C and 95% O_2_, 5% CO_2_ and muscle contractions were elicited through platinum electrodes mounted on either side of the glass chamber and connected to a S48 Stimulator with SUI5 Stimulus Isolation Unit (Grass Technologies, West Warwick, RI, USA). The contractile properties assessed included peak twitch force, maximal isometric tetanic force, time-to-peak twitch force and twitch half-relaxation time, and the maximal rates of tetanic contraction and relaxation.

### 4.4. Histological Analyses of Skeletal Muscle Fibers

Serial 10 µm cross-sections of the GA muscle were acquired and stained first using hematoxylin and eosin for the qualitative analysis of skeletal muscle fibers. Next, muscle sections were stained using NADH-tetrazolium reductase (NADH-TR) and succinate dehydrogenase (SDH) activity. Briefly, to stain for NADH, sections were incubated at 37 °C for 4 min in a solution containing 0.2 M Tris, 1.5 mM NADH, and 1.5 mM NBT [55]. The sections were washed, dehydrated, and cleared in xylenes. SDH staining was conducted by incubating tissues at 37 °C for one hour in a 0.2 M sodium phosphate-buffered solution [56]. The sections were then washed and dehydrated. Hematoxylin and eosin-stained sections were imaged using a brightfield TissueScope LE slide scanner (Huron Digital Pathology, St. Jacobs, ON, Canada) with a 20× objective (0.75 NA, 0.5 µm/pixel resolution). NADH-TR- and SDH-stained sections were imaged using a Nikon Eclipse 200 light microscope at 40× magnification (E Plan 40×/0.65 OFN20) using a digital camera interfaced to Nikon Elements D software (https://www.microscope.healthcare.nikon.com/products/software/nis-elements/nis-elements-documentation, accessed on 26 October 2023) (Nikon Corporation, Tokyo, Japan).

Gastrocnemius muscles were also stained for myosin heavy chain (MyHC) isoform expression. To determine embryonic myosin expression, muscle sections were probed with primary antibodies for sarcomeric (MF20, 5 µg/mL) and embryonic (F1.652, 5 µg/mL) myosin. To classify MyHC isoform expression, muscle sections were probed with MyHC_slow_ (BA-D5, 5 µg/mL) and MyHC_fast_ (F59, 5 µg/mL) primary antibodies. All primary antibodies were acquired from the Developmental Studies Hybridoma Bank (Iowa City, IA, USA). The appropriate corresponding secondary Alex Fluor (Invitrogen, Carlsbad, CA, USA, A21141, A21123, A21240) and DyLight (Jackson ImmunoResearch, West Grove, PA, USA, 115-475-207) antibodies were used at a dilution of 1:200. Fluorescent images were acquired using a Nikon C2 automated upright laser scanning confocal microscope equipped with a Plan Apo λ 40× oil objective and GaASP dual laser detectors (Nikon Instruments Inc., Melville, NY, USA). Image sampling was determined using the Nyquist criterion with a pixel size set to 0.16 µm and dimensions of 2048 × 2048 pixels. During all imaging laser intensity was maintained for each probe.

The gain, offset, gamma, and exposure were constant across samples and probes, and the investigator was blinded to the experimental groups during imaging and analysis. For all histologic outcomes, up to three non-overlapping regions were imaged for each muscle and subsequently exported for analysis. The number of darkly and lightly stained skeletal muscle fibers for the NADH-TR and SDH stains were counted using the multipoint tool for all muscle sections. Darkly stained fibers were categorized as positive for NADH and SDH, signifying high metabolic activity, and lightly stained fibers were classified as negative, indicating low metabolic activity. The fibers of the gastrocnemius were classified individually as embryonic based on the expression of embryonic staining and are presented as a percentage of total fibers. Fibers were classified individually as type I or type II based on the expression of MyHC_slow_ or MyHC_fast_, respectively.

### 4.5. Microscopic Evaluation of Muscle Structure

To image the TA muscle samples, we used a homebuilt 2-photon microscope that was described previously [57]. We tuned our Ti:Sapphire excitation laser to 775 nm, and then detected emitted SHG signals from myosin/actin structures using a 390/18 filter, AlexaFluor 488 (Thermo Fisher Scientific, Waltham, MA, USA) signals using a 525/50 nm bandpass filter, and MitoSpy Orange signals using a 585/40 nm bandpass filter. SHG images were used to measure spacing between the sarcomeres by drawing a line profile perpendicular to the myosin filament and measuring distances between the myosin filament peak intensities. To visualize the α-tubulin network, we fixed TA muscles with 4% PFA for 48 h while rotating, then washed and blocked the muscle with BSA. We incubated the muscle with the anti-α-tubulin overnight, followed by another wash and blocking step. We then allowed the muscle to incubate with an AlexaFluor 488 antibody for an hour, washed the muscle, and mounted it on a slide using Vectashield, followed by imaging. To image the mitochondrial network, the TA muscle was fixed with 4% PFA for 10 min while rotating, followed by a PBS wash, 1% Triton permeabilization for 15 min while rotating, and washed again. We then incubated the muscle with 250 nM MitoSpy Orange and allowed it to rotate for 30 min before a final wash and mounting using Vectashield (Vector Laboratories, Inc., Newark, CA, USA) before imaging.

### 4.6. Mitochondrial Oxygen Consumption

Mitochondrial function was assessed through oxygen consumption rates obtained by high resolution respirometry (Oroboros O2k) of permeabilized muscle fiber bundles. Due to the small muscle size of P14 mice, both TA muscles were dissected into fiber bundles, pooled together, and permeabilized with 50 μg/μL of saponin as previously described [58]. Approximately 2 mg of permeabilized fibers were then weighed, loaded into each O2k chamber, and given time to equilibrate to a baseline respiration rate before the addition of respiration substrates. Complex I leak respiration was accomplished by the addition of glutamate (10 mM) and malate (5 mM), followed by complex I- and II-derived leak respiration with the addition of succinate (10 mM). State III respiration was accomplished by adding ADP (5 mM) and mitochondrial membrane integrity was checked by the addition of cytochrome c (10 µM) after state III respiration was recorded. Any test where cytochrome c addition resulted in a >10% increase over the state III respiration rate was excluded from further analysis. Lastly, FCCP (1 µM) was added to obtain an uncoupled (maximum capacity) respiration rate. Baseline respiration was subtracted out and respiration state rates were normalized to the wet weight of the muscle fiber bundles added to the chamber.

### 4.7. Mitochondrial Content (Citrate Synthase Activity)

Citrate synthase enzyme activity was assessed to quantify mitochondrial content. The remaining portion of the TA muscle fibers not used in the O2k measurements were immediately homogenized in 33 mM phosphate buffer (pH 7.4) at a muscle to buffer ratio of 1:40 using a glass tissue grinder. Citrate synthase activity was measured using a spectrophotometer to detect the reduction of DTNB over time as previously described [59].

### 4.8. Phosphocreatine Creatine Kinase Clamp Respiration

The phosphocreatine creatine kinase clamp technique was performed according to the methodology described by Fisher-Wellman et al. [26] and validated in our hands [28]. This technique utilizes sequential titers of phosphocreatine to modulate ATP/ADP ratios within the closed O2k bath, allowing for the assessment of mitochondrial respiration (*J*O_2_) across a range of ATP free-energy states (ΔG_ATP_). All experiments were carried out at 30 °C in a 2 mL reaction volume. All assays used Buffer Z supplemented with ATP (5 mM), creatine (5 mM), phosphocreatine (PCr, 1 mM), and creatine kinase (20 U/mL). For each experiment, permeabilized skeletal muscle fiber bundles (2 mg) from the TA muscle of HPP and WT animals were suspended in the chamber and supplemented with oxygen before the addition of respiratory substrates. To assess the metabolic flexibility of complexes I and II, the following conditions were tested: Complex I—glutamate/malate (Glu/Mal; 10/2.5 mM) and succinate/rotenone (Succ/R; 10/0.005 mM). Following substrate addition, titrations of PCr (1, 2, 4,7, 16, and 31 mM) were performed to reduce oxygen consumption back to baseline. G’_ATP_ was calculated for each PCr titration and plotted against *J*O_2_ for each step. The resulting slope for each sample (ΔG_ATP_ vs. *J*O_2_) represents the electron conductance of the respiratory system under each condition.

### 4.9. Statistical Analysis

Statistical analysis was performed using Matlab software (version R2020a Update 5 (9.8.0.1451342); The MathWorks Inc., Natick, MA, USA) or JMP Pro statistical software (version 14.2, SAS Institute, Cary, NC, USA). Independent *t*-tests assessed mean differences between WT and HPP genotypes for quantitative analyses. Data are presented as mean ± standard deviation (SD). The statistical significance level was set at *p* ≤ 0.05.

## Figures and Tables

**Table 1:** HPP mouse health and growth metrics for this study (mean ± standard deviation).

Postnatal Day	HPP Weight	HPP Weight as Percent of WT Littermate	HPP Body Condition Score	Abnormal Movement/HPP Total
P10	4.67 ± 0.82 g	84.8 ± 18.2%	2.63 ± 0.34	3/31
P12	5.16 ± 0.88 g	84.3 ± 16.3%	2.61 ± 0.34	6/31
P14	5.58 ± 0.99 g	87.6 ± 27.6%	2.64 ± 0.33	11/31

## Data Availability

Data is available on request.
